# Obesity: A Global Health Challenge Demanding Urgent Action

**DOI:** 10.3390/biomedicines13020502

**Published:** 2025-02-18

**Authors:** Zaida Abad-Jiménez, Teresa Vezza

**Affiliations:** 1 Department of Endocrinology and Nutrition, Doctor Peset University Hospital—Foundation for the Promotion of Health and Biomedical Research in the Valencian Region (FISABIO), 46017 Valencia, Spain; 2 Digestive System Service, Virgen de las Nieves University Hospital, 18014 Granada, Spain; 3 Instituto de Investigación Biosanitaria (ibs.GRANADA), 18012 Granada, Spain

Obesity has become one of the most critical health crises of the modern era, affecting millions of individuals worldwide. In 2021, the World Health Organization (WHO) estimated that over 1 billion individuals were living with obesity, including approximately 650 million adults, 340 million adolescents, and 39 million children [1]. This complex, chronic disease has reached epidemic proportions, significantly contributing to the growing burden of non-communicable diseases. Far from being merely a condition of excess weight, obesity is a central driver of numerous severe health complications, including cardiovascular diseases, type 2 diabetes (T2D), hyperlipidemia, and an elevated risk of premature mortality [2].

The etiology of obesity is multifactorial, involving a dynamic interplay of genetic predispositions, environmental influences, and behavioral factors. Sedentary lifestyles, high-calorie diets, socioeconomic disparities, and urbanization have further exacerbated its prevalence. Equally pivotal are genetic factors, which determine individual susceptibility to obesity and its associated metabolic consequences [3]. These genetic variations help explain why some individuals develop obesity in obesogenic environments, while others remain unaffected [4]. Thus, understanding these factors is critical for advancing personalized preventive and therapeutic strategies. Moreover, disparities in obesity prevalence and its associated risks across demographic groups underscore the need for targeted interventions. For example, non-Hispanic White individuals and Black individuals display varying susceptibilities to obesity and related complications, such as T2D, highlighting the intricate interplay between genetic predispositions and social determinants of health [5,6].

Recent findings published in the Special Issue entitled “Molecular Research in Obesity” of *Biomedicines* provide crucial insights into the multifaceted nature of obesity. Highlights include the identification of specific genetic variants associated with increased risk, as well as novel biomarkers that could serve as targets for early intervention. Moreover, new research underscores the significant role of adipose tissue dysfunction in driving chronic inflammation and metabolic disturbances, offering potential therapeutic pathways for restoring energy homeostasis and reducing the burden of obesity-related comorbidities.

In addition, recent studies in this Special Issue also underscore the intricate relationship between the gut microbiota and obesity. A particularly intriguing study published shows that obesity can be linked to decreased levels of vitamin B12 [7], a micronutrient primarily synthesized by gut microbes. The researchers found that obese mice had significantly fewer enzymes involved in the biosynthesis of vitamin B12 compared to non-obese control. This enzyme reduction was even more pronounced in genetically obese mice, suggesting that the composition of the microbiome plays a key role in regulating vitamin B12 synthesis. As the researchers concluded, “the degree of obesity and the composition of the microbiota are the main factors influencing the expression of genes and pathways for vitamin B12 biosynthesis in the gut”, emphasizing the potential for microbiome-based approaches to address nutrient deficiencies associated with obesity [7].

In a complementary approach, other studies in this Special Issue have examined the effects of postbiotics derived from *Lacticaseibacillus paracasei* strains on adipocyte metabolism [8]. These strains, isolated from *Formica rufa anthills*, demonstrated promising results in improving glucose uptake and enhancing lipid turnover in mature 3T3-L1 adipocytes without promoting excessive lipid accumulation. Among them, the P4 strain proved particularly effective in increasing the expression of key enzymes involved in beta-oxidation and adiponectin production, such as adipose triglyceride lipase and peroxisomal beta-oxidation enzymes, suggesting its potential to improve metabolic function in individuals with obesity.

Additionally, the role of inflammation in obesity-related metabolic disorders has been extensively studied and is widely recognized as a central factor driving the progression of these conditions. Chronic low-grade inflammation is a hallmark of obesity, contributing to the development of metabolic diseases such as type 2 diabetes, cardiovascular disease, and insulin resistance [9]. The increased fat mass in obesity triggers an inflammatory response that involves immune cells infiltrating adipose tissue, which in turn leads to the release of pro-inflammatory cytokines and adipokines. These molecules disrupt normal metabolic processes, further exacerbating obesity and its associated complications [10].

Several articles in this Special Issue provide novel insights into the molecular mechanisms underlying this inflammatory response, advancing our understanding of how obesity leads to systemic inflammation and metabolic dysfunction. One study in particular sheds light on the predictive role of certain fatty acids in modulating the leptin/adiponectin ratio, a key marker of adipose tissue dysfunction and metabolic imbalance [11]. The researchers identified that the 22:6n3 fatty acid, also known as docosahexaenoic acid (DHA), was strongly correlated with an improved adiponectin/leptin ratio in individuals with severe obesity. This finding is of particular interest because the leptin/adiponectin ratio is an important biomarker for evaluating adipose tissue function and metabolic health. Leptin, a hormone primarily produced by adipocytes, plays a crucial role in regulating energy balance [12], while adiponectin, another adipocyte-derived hormone, has anti-inflammatory and insulin-sensitizing effects [13]. In obese individuals, the elevated levels of leptin and reduced levels of adiponectin contribute to inflammation and insulin resistance [11].

The strong correlation between DHA and the adiponectin/leptin ratio suggests that DHA could serve as a potential predictive marker for inflammation in severe obesity. As an omega-3 polyunsaturated fatty acid, DHA has well-documented anti-inflammatory properties, and its role in modulating the inflammatory response in obesity may help prevent or reduce the metabolic disturbances associated with excessive adiposity [14]. Furthermore, this research paves the way for the development of targeted nutritional interventions that could enhance the intake of DHA or similar fatty acids to improve adipose tissue function and reduce inflammation, ultimately leading to better metabolic outcomes in obese individuals. Supporting this concept, previous studies have highlighted DHA as a potential biomarker for chronic inflammation in obesity. One such study compared eutrophic individuals with those suffering from obesity, revealing that DHA levels in PBMCs were inversely associated with markers of inflammation in obese individuals [15]. These findings reinforce the idea that DHA not only plays a critical role in modulating inflammation but may also serve as a key factor in improving metabolic health in obese individuals.

Another study underscores the intricate connection between obesity-induced inflammation and hypothalamic dysfunction, with a particular focus on the neuropeptide neurosecretory protein GM (NPGM) [16]. This research provides valuable insights into how NPGM-expressing neurons in the hypothalamus play a critical role in regulating lipid metabolism and inflammatory responses, which are key processes in maintaining glucose homeostasis. The hypothalamus, a central brain region that controls energy balance, is increasingly recognized as an important mediator of the systemic metabolic disruptions observed in obesity [17]. In this context, the study suggests that NPGM neurons not only contribute to the regulation of lipid storage but also influence inflammation within the central nervous system, linking metabolic dysfunction with immune responses. The findings indicate that NPGM may act as a key mediator in the communication between the brain and peripheral tissues, modulating both lipid metabolism and the inflammatory state characteristic of obesity. By influencing these processes, NPGM has the potential to play a significant role in the dysregulation of energy homeostasis, contributing to the development of obesity-related metabolic disorders such as insulin resistance and type 2 diabetes [16]. This emerging understanding of NPGM’s involvement in central regulation presents an exciting opportunity for developing new therapeutic strategies aimed at targeting NPGM pathways to control lipid storage and inflammation in the hypothalamus.

In addition to the well-documented roles of hypothalamic dysfunction, alterations in microbiota composition, and systemic inflammation, microRNAs have emerged as key regulators of metabolic health in obesity. MicroRNAs (miRNAs) are small non-coding RNA molecules that modulate gene expression and play a crucial role in various cellular processes, including lipid metabolism, insulin sensitivity, and inflammation [18]. One study in this Special Issue examines the circulating levels of miR-33a [19], a microRNA that is strongly associated with lipid metabolism and is thought to play a role in regulating cholesterol homeostasis and fat storage. The study found that miR-33a was significantly elevated in obese adolescents compared to their non-obese counterparts, suggesting that this microRNA may serve as a biomarker for obesity-related metabolic dysfunction. Furthermore, the study revealed that miR-33a levels correlated with key metabolic parameters, such as insulin resistance and cholesterol levels, further supporting the idea of miR-33a as a predictor of metabolic disturbances in obesity. Interestingly, although exosomal miR-33a expression was lower in obese individuals, it did not show any significant correlation with metabolic measures. This contrasts with the findings for circulating miR-33a, which appear to have stronger links to metabolic dysfunction.

These results highlight the potential of circulating miR-33a as an early indicator of metabolic dysfunction in obese adolescents, potentially identifying those at higher cardiometabolic risk. Thus, these insights enable possibilities for miRNA-based therapeutic strategies that could target specific miRNAs to correct metabolic imbalances in obesity and improve insulin sensitivity while reducing the risk of associated comorbidities.

In a related area of research, recent studies in this Special Issue examine the effects of bariatric surgery on mitochondrial DNA (mtDNA) levels and insulin sensitivity [20]. Bariatric surgery, a procedure aimed at promoting weight loss, has been shown to significantly improve various metabolic parameters, including insulin sensitivity, weight loss, and reductions in comorbidities like T2D and hypertension. However, the precise molecular mechanisms driving these improvements are still not fully understood. The study found that while no significant changes in circulating mitochondrial DNA (cf-mtDNA) levels were observed post-surgery, there were notable improvements in insulin sensitivity and other key metabolic parameters. This suggests that the benefits of bariatric surgery may not be directly related to changes in mitochondrial DNA, but rather to other factors such as alterations in adipose tissue, gut hormones, or the composition of the gut microbiome. In this sense, the gut microbiome plays a crucial role in digestion and metabolic processes, and shifts in its composition after bariatric surgery may influence metabolic health. Interestingly, these findings align with a recent study of healthy adolescents, which found that circulating cf-DNA was more strongly associated with the risk of metabolic syndrome than obesity itself [21].

The authors propose that bariatric surgery may improve metabolic health through mechanisms that do not necessarily involve significant changes in mitochondrial function or mtDNA levels. This perspective challenges the traditional view that mitochondrial dysfunction plays a central role in the pathophysiology of obesity-related metabolic disorders. Instead, the study encourages further exploration into how other molecular pathways, such as those involved in energy regulation, inflammation, and hormonal signaling, contribute to the metabolic improvements observed after bariatric procedures. These findings provide valuable insights into the complex interactions between mitochondria, obesity, and metabolic health, and offer new avenues for research into novel therapeutic approaches for obesity and its associated metabolic disorders.

Lastly, obesity is a significant risk factor for cardiovascular diseases (CVDs), and identifying early biomarkers to predict CVD risk is crucial for improving clinical outcomes and preventing complications. A study featured in this Special Issue suggests that HOMA-IR, a widely used index for assessing insulin resistance, could serve as an effective predictor of plasminogen activator inhibitor-1 (PAI-1) levels, a cytokine strongly associated with both obesity and CVDs [22]. In the study, women with severe obesity displayed altered glycemic parameters, including elevated insulin resistance, which correlated with higher PAI-1 levels. These findings indicate that HOMA-IR could be a valuable marker for identifying individuals at higher cardiovascular risk, especially in the context of obesity. By monitoring insulin resistance and its relationship with PAI-1, clinicians may gain critical insights into metabolic dysfunction and improve early intervention strategies for CVD prevention. Moreover, data from this study revealed a positive correlation between the Atherogenic Index of Plasma (AIP) and Alanine Aminotransferase (ALT), which is consistent with findings from a large-scale study in China involving 7838 participants [23]. The study demonstrated that AIP could serve as an independent predictor for the presence of fatty liver disease. AIP is a ratio calculated from the levels of triglycerides and high-density lipoprotein cholesterol (HDL-C) in the plasma, reflecting the balance between “good” and “bad” cholesterol, which is an important indicator of cardiovascular risk. A higher AIP is often associated with a greater risk of developing heart disease and metabolic disorders. Alanine Aminotransferase (ALT) is an enzyme primarily found in the liver, and elevated levels of ALT typically indicate liver injury or dysfunction. Since ALT is a sensitive marker of liver damage, its levels are often used to assess liver health, including the presence of liver diseases like non-alcoholic fatty liver disease (NAFLD). The positive correlation between AIP and ALT in this study suggests that AIP may not only be a marker for cardiovascular risk but also for liver health, particularly in individuals with obesity. This connection points to AIP as a potentially valuable marker for assessing the risk of NAFLD, which is commonly seen in obese individuals.

The research presented in this Special Issue emphasizes the multifaceted nature of obesity and its complex molecular mechanisms. The studies highlight the promising potential of microbial, inflammatory, and metabolic interventions to address the obesity epidemic. Specifically, they focus on modulating the gut microbiome, targeting therapies for lipid metabolism, and identifying early biomarkers for metabolic dysfunction. However, much remains to be understood about the precise molecular pathways involved in obesity and its related comorbidities.

Looking forward, future research should continue to explore personalized treatment strategies, integrating microbiome analysis, inflammatory markers, and metabolic indicators to create more effective interventions for obesity. The potential of emerging therapeutic approaches, such as targeting neuropeptides like NPGM or enhancing adipose tissue activity, offers exciting possibilities for the management of obesity and its associated disorders. Continued collaboration across disciplines—ranging from microbiology and endocrinology to molecular biology and clinical medicine—will be crucial in unraveling the complexities of obesity and developing novel therapies for this growing public health challenge.

As the Guest Editors of this Special Issue, we are deeply grateful to all the contributors for providing their views of the current developments and future directions in the field of obesity research at the molecular level. These original research articles and reviews span a broad spectrum of topics, from the genetic architecture of obesity to the intricacies of metabolic dysregulation. These contributions not only enhance our understanding of the disease but also inform evidence-based interventions tailored to reduce the global burden of obesity and its related health challenges.

## Data Availability

Data sharing is not applicable to this article because no data sets were generated or analyzed during the present study.

## Abbreviations

DHA, docosahexaenoic acid; HOMA-IR, Homeostasis Model Assessment of Insulin Resistance; mtDNA, mitochondrial DNA; NAFLD, non-alcoholic fatty liver disease; T2D, type 2 diabetes.
